# Exploring the Axis of Gut Microbiota‐Inflammatory Cytokine‐Atrial Fibrillation in the Pathogenesis of Atrial Fibrillation

**DOI:** 10.1111/jcmm.70379

**Published:** 2025-02-10

**Authors:** Jun Chen, Yucheng Wang, Kangnan Wang, Ziwei Mei, Lihong Wang

**Affiliations:** ^1^ Department of Cardiology Zhejiang Provincial People's Hospital, Affiliated People's Hospital, Hangzhou Medical College Zhejiang China; ^2^ Department of Cardiology, Zhongshan Hospital, Shanghai Institute of Cardiovascular Diseases Shanghai Medical College of Fudan University Shanghai China; ^3^ Education Department, Zhejiang Provincial People‘s Hospital Affiliated People‘s Hospital, Hangzhou Medical College Zhejiang China; ^4^ Department of Pharmacy The First Affiliated Hospital of Zhejiang Chinese Medical University, Zhejiang Provincial Hospital of Chinese Medicine Zhejiang China

**Keywords:** atrial fibrillation, gut microbiota, inflammatory cytokine, mediation analysis, mendelian randomization

## Abstract

A relationship may exist between the gut microbiota, inflammatory factors and atrial fibrillation (AF); however, the precise biological mechanisms linking these components remain uncertain.In this study, 211 single‐nucleotide polymorphisms associated with the gut microbiota were collected from the MiBioGen consortium. Summary data for AF were sourced from large‐scale genome‐wide association studies. Two‐step Mendelian randomization (MR) was applied to estimate the possible mediating effect of inflammatory cytokines on the causality between the gut microbiota and AF. MR confirmed the effects of class Lentisphaeria, family Bifidobacteriaceae, family XIII, genus *Anaerostipes*, genus *Howardella*, genus *Intestinibacter*, genus *Lachnospiraceae* (NK4A136 group), genus *Odoribacter*, genus *Ruminococcus gnavus* , order Bifidobacteriales, order Victivallales and phylum Lentisphaerae on AF prevention. Moreover, MR revealed the role of Fms‐related tyrosine kinase 3 ligand, interleukin‐6, interleukin‐7, leukaemia inhibitory factor receptor, sulfotransferase 1A1 and tumour necrosis factor ligand superfamily member 12 in protecting against AF. Fibroblast growth factor 5, interleukin‐2 receptor subunit β, and tumour necrosis factor had a causal effect, increasing AF risk. The mediation exploration indicated that the indirect effect of genus *Lachnospiraceae* (FCS020 group) (id: 11314) on AF mediated by interleukin‐18 was OR 1.015 (95% confidence interval 1.000–1.037; mediation proportion = 9.494%). This study supplies genetic insights into the potential causal association between the gut microbiota and AF. These causal associations and mediating effects are useful for managing AF through manipulation of the gut microbiota.

**Abbreviations**
AF: atrial fibrillation
CI: confidence intervals
CVD: cardiovascular disease
GWAS: genome‐wide association studies
IV: instrumental variables
IVW: inverse variance‐weighted
MR: mendelian randomization
MVMR: multivariable MR
SNP: single nucleotide polymorphism

## 1 Introduction

Atrial fibrillation (AF) is a type of cardiac arrhythmia with a high global prevalence. AF is estimated to affect 0.4% of the general population, equating to around 33.5 million individuals. Age is an independent risk factor for AF, and the incidence of AF is around 6% in adults aged > 65 years [1]. By 2030, it is anticipated that 50 million people will have AF worldwide [2, 3]. Early estimates place the number of people with AF at 17.9 million in Europe and 6–12 million in the United States by 2050 and 2060 [1]. AF is one of the factors contributing to the incidence of cardiovascular and cerebrovascular diseases, and it has a significant financial and health impact on nations across the globe [4].

Several previous studies have explored the risk factors for AF, which include non‐alcoholic fatty liver disease [5], short sleep duration [6], hyperuricemia [7] and frailty [8], among others. Moreover, emerging evidence indicates that the immune system undergoes significant alterations and interacts with the environment and with cells, and these interactions have been implicated in AF [9]. Another study showed that AF exhibits persistent inflammation from immune cell activation and demonstrates proinflammatory cytokine release [10]. These factors stimulate the development of cardiac fibrosis and structural alterations in the atria, such as the deposition of amyloid and collagen and the accumulation of amyloid in the left atrial appendage [11]. However, the precise pathogenesis of AF is still not well understood.

A previous study reported that changes in the gut microbiota affect the heart through the gut–heart axis and disordered gut microbiota is closely related to the development of several cardiovascular diseases [12]. Several observational and Mendelian randomization (MR) studies have observed associations of gut microbiota with AF. For instance, one study showed that compared with the control population, patients with AF showed a significant change in microbial diversity and disordered gut microbiota [13]. Another study showed the varied enrichment of some gut microbiota at different phases of AF, indicating that the gut microbiota influence AF progression. These microbiota included *Butyricicoccus*, *Paraprevotella*, *Blautia*, *Dorea* and *Coprococcus* [14]. In light of this evidence, improving the quality of the gut microbiota should be considered a crucial target in the prediction and treatment of AF. The gut microbiota has a symbiotic association with the host organism and is involved in intestinal immune function. Physiologically, the gut microbiota does not create a harmful immune reaction. However, disordered gut microbiota can exacerbate AF by targeting AF substrates or stimulating AF risk factors [15]. When the gut microbiota is disordered or the intestinal barrier is destroyed, bacteria and metabolites circulate through the bloodstream, leading to a chronic inflammatory response. Inflammatory reactions induced by the gut microbiota are one potential mechanism underpinning AF development. Some researchers have reported that inflammatory cytokines activated by the gut microbiota affect AF progression, and the NLRP3 inflammasome is thought to play a role [16, 17]. Therefore, the causal impact of the gut microbiota and inflammatory cytokines on AF pathogenesis deserves further exploration. This enhanced understanding will provide valuable insights into the prevention of AF.

The construction of instrumental variables (IVs), which are variables that solely impact the result through risk factors, using MR is predicated on genome‐wide association studies (GWAS). With this approach, the confounding factors in observational research can be effectively controlled. Single‐nucleotide polymorphisms (SNPs) are used as IVs in MR analyses to identify risk factors. To imitate the causal associations between risk factors and outcomes, MR takes advantage of the Mendelian regulation of heredity, which suggests that parental alleles are randomly distributed to offspring. Many previous studies have reported the MR results of the gut microbiota in the context of AF [18, 19]. However, these studies only analysed the causal correlations between the gut microbiota and AF. They did not consider the causal associations between inflammatory cytokines and AF and the mediating roles of these inflammatory cytokines in the association between the gut microbiota and AF. In this study, we use summary statistics from the largest and most recent GWAS of the gut microbiota, inflammatory cytokines, and AF to perform two‐step MR and mediation analyses. Our study attempts to shed light on the complex interactions between these components, providing important insights into the causal associations between these variables.

## 2 Materials and Methods

### 2.1 Study Design

This study adopted a two‐step MR design to investigate causality among the gut microbiota, inflammatory cytokines, and AF. First, we considered SNPs in the gut microbiota and inflammatory cytokines as IVs to analyse their causal effects on AF. After determining the qualifying SNPs, we used various analysis techniques, including inverse variance‐weighting (IVW), weighted mode, MR‐Egger regression, weighted median. The stability of the results was checked by performing sensitivity analyses containing Cochran's *Q* test, the MR‐Egger intercept test, and the leave‐one‐out analysis. Second, we analysed the causal impact of AF on the gut microbiota. Third, we evaluated the causal impact of the gut microbiota on inflammatory cytokines. Finally, we calculated the combined causal effect of the gut microbiota and inflammatory cytokines on AF based on multivariable MR (MVMR) and mediation effect analyses. The methodology of this MR study adhered to the standards of the STrengthening the Reporting of OBservational studies in Epidemiology using MR (STROBE‐MR) statement [20].

### 2.2 Data Sources

#### 2.2.1 Gut Microbiota Samples

A comprehensive meta‐analysis of multi‐ethnic GWAS was performed to derive summary statistics for gastrointestinal microbial taxa. This analysis encompassed 18,340 individuals across 24 cohorts [21]. Three different variable sections of the *16S* rRNA gene were used to analyse the microbial composition. All datasets were filtered to 10,000 reads per sample to compensate for variations in the sequencing depth. Direct taxonomic binning was used for taxonomic classification. Only those found in over 10% of the samples in each cohort were fitted to investigate the impact of host genetics on the richness of the gut bacterial taxa. The inclusion in at least three cohorts and an effective sample size of 3000 people were the study‐wide cutoffs. There were 211 taxa comprising 131 genera, 35 families, 20 orders, 16 classes, and 9 species. Spearman's correlation analysis was used to identify the genetic loci that influenced the covariate‐adjusted richness of the bacterial taxa after controlling for age, sex, technical variables, and genetic principal components. More information on the microbiome data has been published previously [21].

### 2.3 Data Source for Circulating Inflammatory Cytokines

The present study utilised data on inflammatory cytokines from the GWAS catalogue database (ID: GCST90274758‐GCST90274848) and the University of Bristol (https://data.bris.ac.uk/data/dataset) [22].

### 2.4 AF Samples

A comprehensive investigation of over 10,00,000 individuals yielded summary GWAS data for AF, comprising 60,620 cases and 970,216 controls [23]. The present study identified 142 distinct risk variants at 111 loci and preferentially processed 151 functional candidate genes believed to play a role in AF.

### 2.5 IV Selection

At six taxonomic levels, the 211 bacterial taxa were grouped. The genus is the lowest and most specialised taxonomic rank. We only evaluated 131 bacterial taxa from the genus perspective to pinpoint each causative bacterial group as precisely as possible. Overall, 117 bacterial taxa were incorporated in the ensuing MR analysis after removing 14 taxa with unclear groups.

MR makes three assumptions: (1) the SNPs are significantly correlated with the exposure; (2) the SNPs are not related to confounders of the outcome; and (3) the SNPs impact the outcome only through the exposure. To satisfy the first assumption, we identified candidate IVs as SNPs linked to the gut bacterial taxa according to the genome‐wide significance criterion *p* < 1.0 × 10^−5^. We identified the risk factors for AF according to the reported evidence. After searching the information of each SNP on the PhenoScanner V2 website (http://www.phenoscanner.medschl.cam.ac.uk/), we deleted SNPs associated with confounders of AF and with AF to target the last two assumptions.

Eligible IVs were chosen by quality control processes. First, SNPs with different alleles between the exposure and outcome (A/G vs. A/C) were omitted. Second, palindromic A/T or G/C alleles were omitted. Third, only independent SNPs were retained by clumping the SNPs inside each bacterial taxon. The size of the clumping window was 1000 kb, and the linkage disequilibrium (LD) criterion for clumping was set at *R* ^2^ < 0.001. The LD was evaluated using the reference panel of the 1000 Genome Project based in Europe. Finally, the MR Pleiotropy RESidual Sum and Outlier (MR‐PRESSO) test was applied to identify possible horizontal pleiotropy and eradicate its effects by excluding outliers [24].

In accordance with a previous publication, the *R* ^2^ and F statistic of each SNP were computed in the contained exposure cohort (the IV) [25]. The formula used to calculate *R* ^2^ was as follows: *R* ^2^ = 2 × minor allele frequency (MAF) × (1 − MAF) × beta.exposure^2^, where *R* ^2^ is the percentage of variation in the instrument that is explained. The formula for calculating the *F*‐statistic was as follows: *F* = beta.exposure^2^ ÷ standard error.exposure^2^.

### 2.6 Statistical Analysis

The association between the gut microbiota and sepsis was evaluated using a bidirectional MR analysis. The reliable MR analysis technique of IVW was used for our primary meta‐analysis [26]. Additionally, we conducted secondary analyses by MR‐Egger regression [27] and weighted median [28]. We assessed the possible effects of directed pleiotropy by evaluating the intercept value of MR‐Egger regression [27], and we evaluated heterogeneity using Cochran's *Q* test [24]. When heterogeneity existed, we ran the primary analysis using random‐effects IVW. At every characteristic level (phylum = 9, class = 15, order = 19, family = 30, genus = 117), we applied a multiple‐testing statistical criterion of *p* < 0.05/*n*, where *n* is the efficient amount of distinct bacterial taxa from the pertinent taxonomic view.

For the mediation aspect of the study, univariable MR was used to estimate the overall influence of a risk factor on the outcome. Two‐step MR and MVMR were used to distinguish between direct and indirect effects. Using univariable MR, we first assessed how the exposure affected the mediator. In the second step, MVMR was used to estimate the impact of the mediator on each outcome. Univariable MR has been recommended for determining the influence of the mediator in place of MVMR, which has not been used in previous research for this second step [29, 30]. However, in the case of MVMR, it is guaranteed that the impact of the mediator on the result operates independently of the exposure's influence [31]. Moreover, the exposure is directly impacted by the mediator. Multiplying the two‐step MR estimations yields the indirect effect estimate. The exposures and mediators with true effects were identified using stepwise regression [32, 33].

## 3 Results

### 3.1 Two‐Sample and MR Analyses of the Gut Microbiota and AF Risk

**FIGURE 1:**
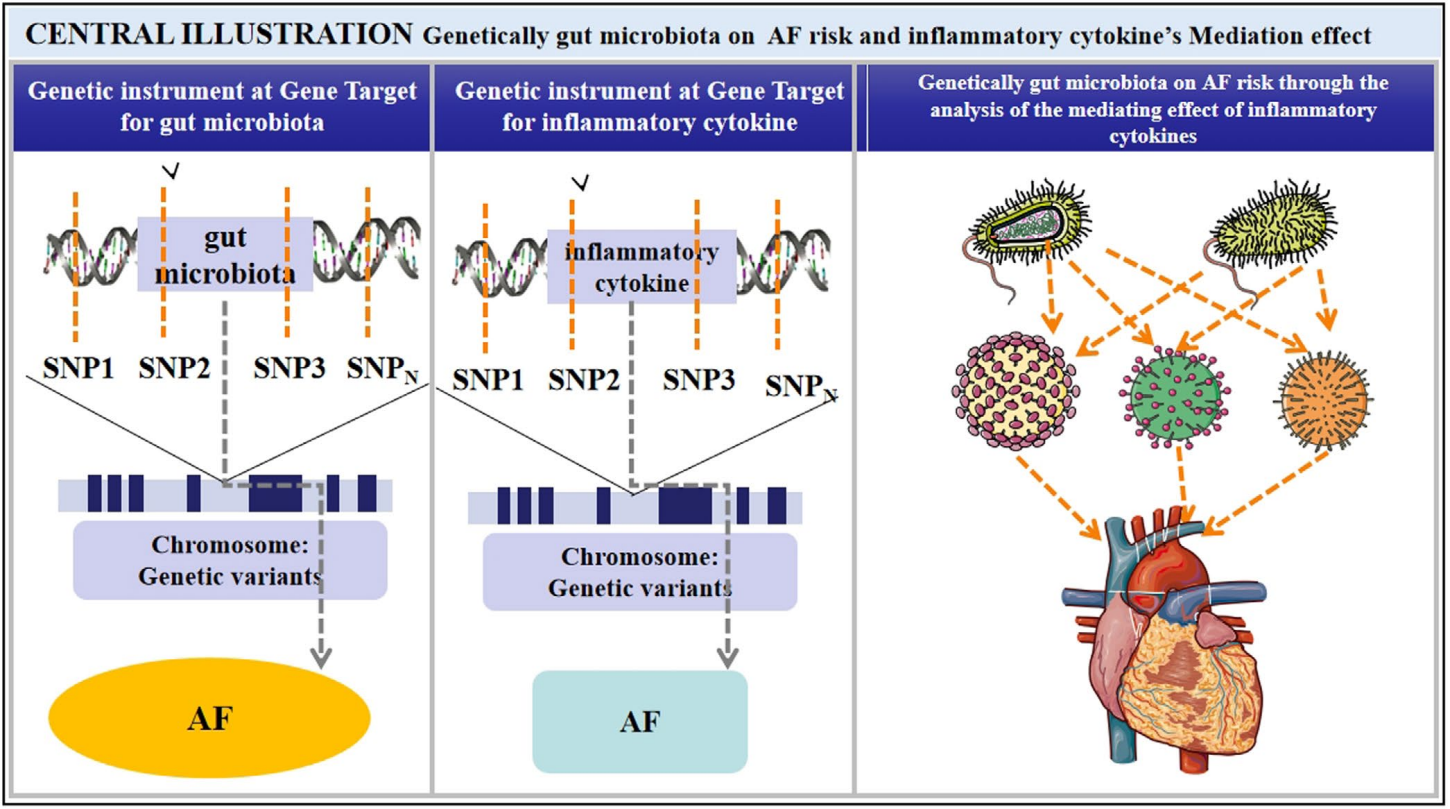
Flowchart illustrating the study selection process.

**FIGURE 2:**
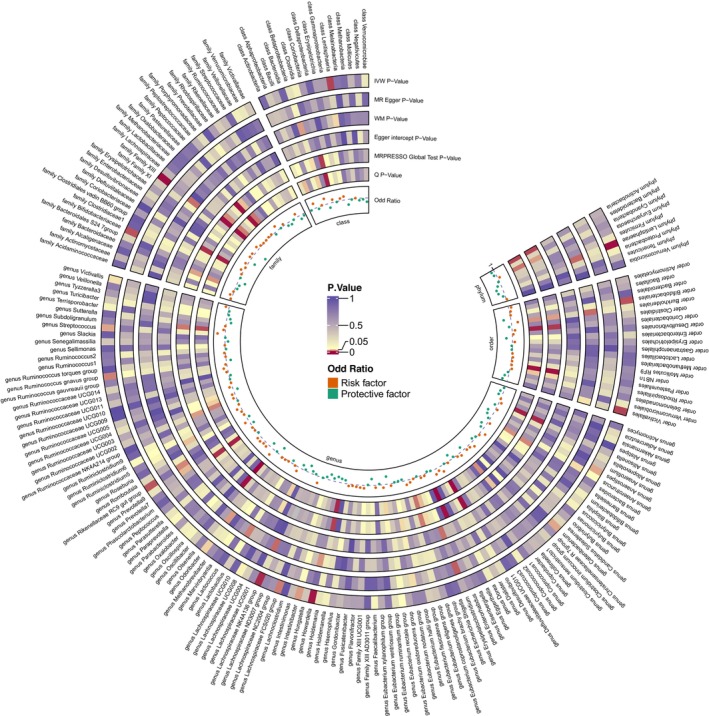
Annular heatmap. Preliminary MR analyses of the associations between the gut microbiota and AF risk. The circles from the outer to the inner represent the IVW, MR‐Egger regression, weighted median and weighted mode methods, respectively. The shades of colour reflect the magnitude of the *p* value. IVW, inverse variance‐weighted; MR, mendelian randomization; MR‐Egger, MR egger regression.

The overall study design is depicted in Figure 1. The data included in this study are shown in Table 1, and Figure 2 illustrates the aggregate outcomes. The impact of the gut microbiota was explored by TSMR analysis, which indicated that class Lentisphaeria exerted a protective role against AF (odds ratio [OR] 0.939 per standard deviation [SD] increase in class Lentisphaeria, 95% CI 0.896–0.984; *p* = 0.008), as did family Bifidobacteriaceae (OR 0.916 per SD increase, 95% CI 0.855–0.980; *p* = 0.012), family XIII (OR 0.867 per SD increase, 95% CI 0.799–0.940; *p* = 0.001), genus *Anaerostipes* (OR 0.922 per SD increase, 95% CI 0.857–0.993; *p* = 0.031), genus *Howardella* (OR 0.939 per SD increase, 95% CI 0.904–0.977; *p* = 0.002), genus *Intestinibacter* (OR 0.933 per SD increase, 95% CI 0.877–0.993; *p* = 0.028), genus *Lachnospiraceae* NK4A136 group (OR 0.918 per SD increase, 95% CI 0.865–0.973; *p* = 0.004), genus *Odoribacter* (OR 0.909 per SD increase, 95% CI 0.831–0.996; *p* = 0.040), genus *Ruminococcus gnavus* group (OR 0.948 per SD increase, 95% CI 0.906–0.992; *p* = 0.022), order Bifidobacteriales (OR 0.916 per SD increase, 95% CI 0.855–0.980; *p* = 0.012), order Victivallales (OR 0.939 per SD increase, 95% CI 0.896–0.984; *p* = 0.008), and phylum Lentisphaerae (OR 0.926 per SD increase, 95% CI 0.886–0.968; *p* = 0.001).

**TABLE 1:** Summary of genome‐wide association studies included in this study.

Phenotype	Cohort (s)	Sample size	Race	GWAS data source
Gut microbial	large‐scale multi‐ethnic GWAS meta‐analysis 24 cohorts	18,340	European	Kurilshikov A, Large‐scale association analyses identify host factors influencing human gut microbiome composition. Nat Genet. 2021
Inflammatory cytokines	University of Bristol	23,363 (ncase) 187,840 (ncontrol)	European	Zhao JH, Genetics of circulating inflammatory proteins identifies drivers of immune‐mediated disease risk and therapeutic targets. Nat Immunol. 2023. (ID: ebi‐a‐GCST90016908‐ebi‐a‐GCST90017118)
AF	European population	60,620 (ncase) 970,216 (ncontrol)	European	Nielsen JB, Biobank‐driven genomic discovery yields new insight into atrial fibrillation biology. Nat Genet. 2018. (ebi‐a‐GCST006414)

We also found that genus *Lachnospiraceae* UCG008 (OR 1.051 per SD increase, 95% CI 1.001–1.104; *p* = 0.047), genus *Rikenellaceae* RC9 (OR 1.047 per SD increase, 95% CI 1.010–1.086; *p* = 0.012), and genus *Streptococcus* (OR 1.089 per SD increase, 95% CI 1.008–1.177; *p* = 0.030) had causative effects on AF, increasing its risk.

**FIGURE 3:**
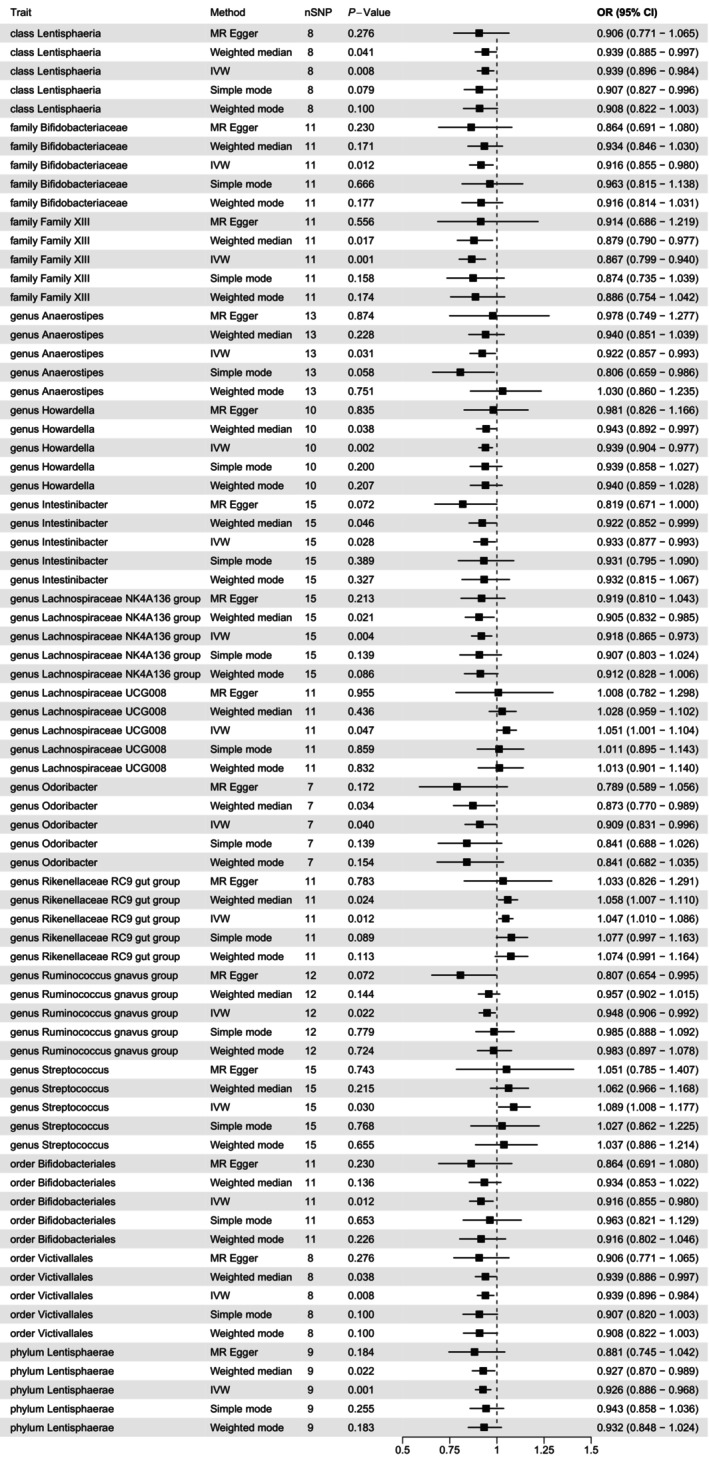
Forest plot of the causal effect of the gut microbiota on AF. AF, atrial fibrillation.

The MR results are shown in Figure 3 and Table 2. Overall, TSMR confirmed the protective effects of 12 gut microbes against AF development, including Lentisphaeria, Bifidobacteriaceae, family XIII, *Anaerostipes*, *Howardella*, *Intestinibacter*, *Lachnospiraceae* NK4A136, *Odoribacter*, *Ruminococcus gnavus* , Bifidobacteriales, Victivallales and Lentisphaerae. Three gut microbes increased the risk of AF, including *Lachnospiraceae* UCG008, *Rikenellaceae* RC9, and *Streptococcus*. More details of these results were presented in Table S1.

**TABLE 2:** The causal relationship of gut microbes and AF.

AF	Gut microbes	OR	95% CI	*p*
—	Gut microbiota abundance (class Alphaproteobacteria id.2379)	0.947	0.908–0.987	0.010
—	Gut microbiota abundance (genus *Faecalibacterium* id.2057)	0.965	0.936–0.994	0.018
—	Gut microbiota abundance (genus *Actinomyces* id.423)	0.949	0.906–0.994	0.027
—	Gut microbiota abundance (family Rhodospirillaceae id.2717)	0.954	0.913–0.996	0.033
—	Gut microbiota abundance (genus *Ruminiclostridium5* id.11355)	0.965	0.933–0.997	0.034
—	Gut microbiota abundance (family *Veillonellaceae* id.2172)	0.966	0.936–0.997	0.036
—	Gut microbiota abundance (class Negativicutes id.2164)	0.970	0.942–0.999	0.047
—	Gut microbiota abundance (order Selenomonadales id.2165)	0.970	0.942–0.999	0.047
—	Gut microbiota abundance (family Defluviitaleaceae id.1924)	1.054	1.008–1.101	0.020
—	Gut microbiota abundance (genus *Defluviitaleaceae* UCG011 id.11287)	1.051	1.006–1.099	0.027
—	Gut microbiota abundance (genus *Erysipelatoclostridium* id.11381)	1.046	1.004–1.089	0.031

### 3.2 TSMR Analysis of Inflammatory Cytokines and AF Risk

The TSMR analysis revealed the function of inflammatory cytokines. The results suggest that the CD40L receptor protected against AF (OR 0.964 per SD increase in expression, 95% CI 0.934–0.995; *p* = 0.023), as did Fms‐related tyrosine kinase 3 ligand (OR 0.954 per SD increase, 95% CI 0.917–0.991; *p* = 0.017), interleukin‐6 (OR 0.886 per SD increase, 95% CI 0.788–0.997; *p* = 0.044), interleukin‐7 (OR 0.927 per SD increase, 95% CI 0.866–0.994; *p* = 0.032), leukaemia inhibitory factor receptor (OR 0.927 per SD increase, 95% CI 0.872–0.986; *p* = 0.017), sulfotransferase 1A1 (OR 0.946 per SD increase, 95% CI 0.909–0.985; *p* = 0.007), and tumour necrosis factor ligand superfamily member 12 (OR 0.916 per SD increase, 95% CI 0.868–0.968; *p* = 0.002).

We also found that fibroblast growth factor 5 (OR 1.072 per SD increase in concentration; 95% CI 1.046–1.099; *p* < 0.001), interleukin‐2 receptor subunit β (OR 1.072 per SD increase, 95% CI 1.005–1.143; *p* = 0.036), and tumour necrosis factor (OR 1.089 per SD increase, 95% CI 1.030–1.152; *p* = 0.003) had causative effects, increasing the risk of AF.

**FIGURE 4:**
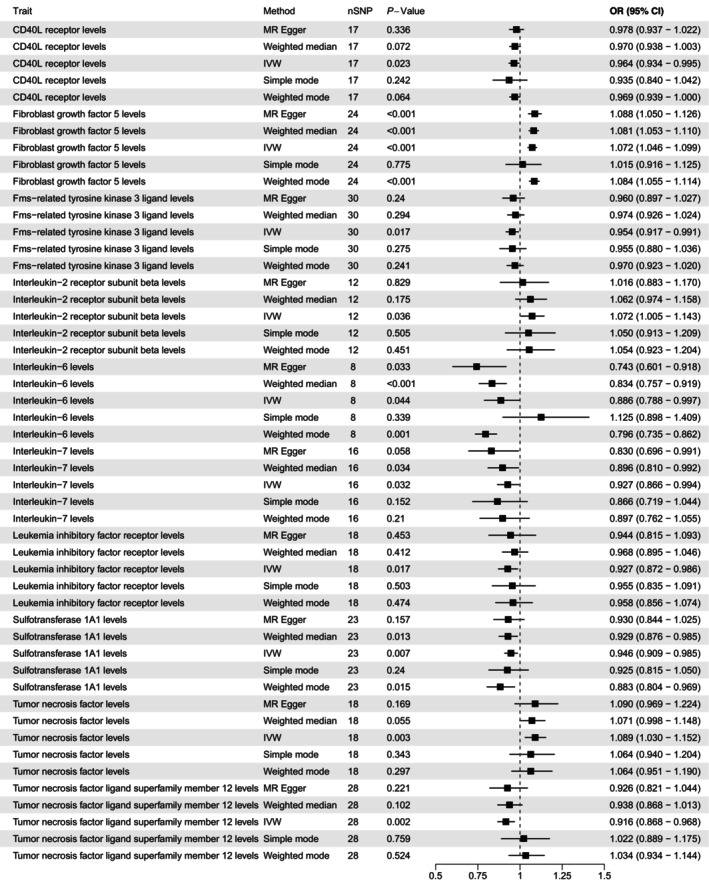
Forest plot of the causal effect of inflammatory cytokines on AF. AF, atrial fibrillation.

The MR results are shown in Figure 4. TSMR revealed six inflammatory cytokines with protective effects against AF, including Fms‐related tyrosine kinase 3 ligand, interleukin‐6, interleukin‐7, leukaemia inhibitory factor receptor, sulfotransferase 1A1 and tumour necrosis factor ligand superfamily member 12. Conversely, fibroblast growth factor 5, interleukin‐2 receptor subunit β and tumour necrosis factor increased the risk of AF. More details of these results were presented in Table S2.

### 3.3 Causal Relationship Between the Gut Microbiota and Inflammatory Cytokines

The increased abundance of genus *Intestinibacter* (id: 11345) had a causal effect on the increased serum concentration of fibroblast growth factor 5 (OR 1.129, 95% CI 1.013–1.258; *p* = 0.029), tumour necrosis factor (OR 1.126, 95% CI 1.009–1.256; *p* = 0.035), Fms‐related tyrosine kinase 3 ligand (OR 1.087, 95% CI 1.002–1.179; *p* = 0.044), and interleukin‐2 receptor subunit β (OR 1.116, 95% CI 1.000–1.244; *p* = 0.049). The increased abundance of family Bifidobacteriaceae (id: 433) had a causal effect on the increased serum concentration of fibroblast growth factor 5 (OR 1.230, 95% CI 1.084–1.397; *p* = 0.001) and interleukin‐2 receptor subunit β (OR 1.139, 95% CI 1.001–1.295; *p* = 0.049). The increased abundance of order Bifidobacteriales (id: 432) had a causal effect on the increased serum concentration of fibroblast growth factor 5 (OR 1.230, 95% CI 1.084–1.397; *p* = 0.001) and interleukin‐2 receptor subunit β (OR 1.139, 95% CI 1.001–1.295; *p* = 0.049). The increased abundance of genus *Rikenellaceae* RC9 gut group (id: 11191) had a causal effect on the decreased serum concentration of sulfotransferase 1A1 (OR 0.916, 95% CI 0.861–0.975; *p* = 0.006) and interleukin‐6 (OR 0.917, 95% CI 0.854–0.984; *p* = 0.017). The increased abundance of genus *Streptococcus* (id: 1853) had a causal effect on the decreased serum concentration of interleukin‐7 (OR 0.835, 95% CI 0.745–0.937; *p* = 0.002). The increased abundance of genus *Lachnospiraceae* NK4A136 group (id: 11319) had a causal effect on the decreased serum concentration of tumour necrosis factor (OR 0.878, 95% CI 0.783–0.985; *p* = 0.027). The increased abundance of genus *Howardella* (id: 2000) had a causal effect on the decreased serum concentration of tumour necrosis factor (OR 0.924, 95% CI 0.856–0.997; *p* = 0.041). The increased abundance of genus *Intestinibacter* (id: 11345) had a causal effect on the increased serum concentration of Fms‐related tyrosine kinase 3 ligand (OR 1.087, 95% CI 1.002–1.179; *p* = 0.044), and the increased abundance of phylum Lentisphaerae (id: 2238) had a causal effect on the increased serum concentration of Fms‐related tyrosine kinase 3 ligand (OR 1.081, 95% CI 1.000–1.168; *p* = 0.049). The MR results are shown in Table 3. More details of these results were presented in Table S3.

**TABLE 3:** The causal relationship of gut microbes to inflammatory proteins.

Gut microbes	Inflammatory proteins	OR	95% CI	*p*
Gut microbiota abundance (genus *Intestinibacter* id.11345)	Fibroblast growth factor 5 levels	1.129	1.013–1.258	0.029
Gut microbiota abundance (genus *Intestinibacter* id.11345)	Tumour necrosis factor levels	1.126	1.009–1.256	0.035
Gut microbiota abundance (genus *Intestinibacter* id.11345)	Fms‐related tyrosine kinase 3 ligand levels	1.087	1.002–1.179	0.044
Gut microbiota abundance (genus *Intestinibacter* id.11345)	Interleukin‐2 receptor subunit beta levels	1.116	1.000–1.244	0.049
Gut microbiota abundance (family Bifidobacteriaceae id.433)	Fibroblast growth factor 5 levels	1.230	1.084–1.397	0.001
Gut microbiota abundance (family Bifidobacteriaceae id.433)	Interleukin‐2 receptor subunit beta levels	1.139	1.001–1.295	0.049
Gut microbiota abundance (order Bifidobacteriales id.432)	Fibroblast growth factor 5 levels	1.230	1.084–1.397	0.001
Gut microbiota abundance (order Bifidobacteriales id.432)	Interleukin‐2 receptor subunit beta levels	1.139	1.001–1.295	0.049
Gut microbiota abundance (genus *Rikenellaceae* RC9 gut group id.11191)	Interleukin‐6 levels	0.916	0.861–0.975	0.006
Gut microbiota abundance (genus *Rikenellaceae* RC9 gut group id.11191)	Sulfotransferase 1A1 levels	0.917	0.854–0.984	0.017
Gut microbiota abundance (genus *Ruminococcus gnavus* group id.14376)	Sulfotransferase 1A1 levels	1.152	1.053–1.261	0.002
Gut microbiota abundance (genus *Streptococcus* id.1853)	Interleukin‐7 levels	0.835	0.745–0.937	0.002
Gut microbiota abundance (genus *Lachnospiraceae* NK4A136 group id.11319)	Tumour necrosis factor levels	0.878	0.783–0.985	0.027
Gut microbiota abundance (genus *Howardella* id.2000)	Tumour necrosis factor levels	0.924	0.856–0.997	0.041
Gut microbiota abundance (order Victivallales id.2254)	Fms‐related tyrosine kinase 3 ligand levels	1.087	1.002–1.179	0.044
Gut microbiota abundance (phylum Lentisphaerae id.2238)	Fms‐related tyrosine kinase 3 ligand levels	1.081	1.000–1.168	0.049

### 3.4 Mediation Analysis

The mediation analysis showed that the indirect effect of genus *Lachnospiraceae* FCS020 group (id: 11314) on AF mediated by interleukin‐18 concentration was OR 1.015 (95% CI 1.000–1.037, mediation proportion = 9.494%) (Table 4).

**TABLE 4:** The results of the mediation analysis.

Gut microbes	Mediator	Mediation effect (*β* (95% CI))	OR (95% CI)	Proportion of mediation effect
Gut microbiota abundance (genus *Intestinibacter* id.11345)	Fms‐related tyrosine kinase 3 ligand levels	−0.005 (−0.012–0.000)	0.995 (0.988–1.000)	—
Gut microbiota abundance (genus *Intestinibacter* id.11345)	Fibroblast growth factor 5 levels	0.009 (0.001–0.018)	1.009 (1.001–1.018)	—
Gut microbiota abundance (genus *Intestinibacter* id.11345)	Interleukin‐2 receptor subunit beta levels	0.007 (−0.001–0.020)	1.007 (0.999–1.021)	—
Gut microbiota abundance (genus *Intestinibacter* id.11345)	Tumour necrosis factor levels	0.005 (−0.003–0.018)	1.005 (0.997–1.018)	—
Gut microbiota abundance (family Bifidobacteriaceae id.433)	Fibroblast growth factor 5 levels	0.014 (0.005–0.026)	1.014 (1.005–1.026)	—
Gut microbiota abundance (family Bifidobacteriaceae id.433)	Interleukin‐2 receptor subunit beta levels	0.007 (−0.003–0.022)	1.007 (0.997–1.022)	—
Gut microbiota abundance (genus *Howardella* id.2000)	Tumour necrosis factor levels	−0.003 (−0.010–0.003)	0.997 (0.990–1.003)	—
Gut microbiota abundance (genus *Lachnospiraceae* NK4A136 group id.11319)	Tumour necrosis factor levels	−0.001 (−0.011–0.005)	0.999 (0.990–1.005)	—
Gut microbiota abundance (genus *Rikenellaceae* RC9 gut group id.11191)	Interleukin‐6 levels	0.011 (0.002–0.024)	1.011 (1.002–1.024)	23.913%
Gut microbiota abundance (genus *Rikenellaceae* RC9 gut group id.11191)	Sulfotransferase 1A1 levels	0.004 (0.000–0.009)	1.004 (1.000–1.010)	—
Gut microbiota abundance (genus *Ruminococcus gnavus* group id.14376)	Sulfotransferase 1A1 levels	−0.004 (−0.012–0.001)	0.996 (0.988–1.001)	—
Gut microbiota abundance (genus *Streptococcus* id.1853)	Interleukin‐7 levels	0.009 (−0.005–0.027)	1.009 (0.995–1.027)	—
Gut microbiota abundance (order Bifidobacteriales id.432)	Fibroblast growth factor 5 levels	0.014 (0.005–0.026)	1.014 (1.005–1.026)	—
Gut microbiota abundance (order Bifidobacteriales id.432)	Interleukin‐2 receptor subunit beta levels	0.007 (−0.003–0.022)	1.007 (0.997–1.022)	—
Gut microbiota abundance (order Victivallales id.2254)	Fms‐related tyrosine kinase 3 ligand levels	−0.005 (−0.012–0.000)	0.995 (0.988–1.000)	—
Gut microbiota abundance (phylum Lentisphaerae id.2238)	Fms‐related tyrosine kinase 3 ligand levels	−0.004 (−0.011–0.000)	0.996 (0.989–1.000)	—

## 4 Discussion

MR is a methodology that uses genetic diversity to explore the underlying causality between exposures and outcomes. In this study, we applied MR to identify 12 gut microbes that genetically reduce the risk of AF and three that are associated with an increase in AF risk, with unidirectional causality. Moreover, the mediation analysis uncovered the mediating role of interleukin‐18 on the causality between the genus *Lachnospiraceae* FCS020 group (id: 11314) and AF. This study unveils the causal association between the gut microbiota and AF, stressing the mediation effect of inflammatory cytokine concentrations. Our findings suggest that gut microbiota‐induced inflammatory cytokines should be considered as part of risk stratification for AF.

The gut microbiota has a crucial impact on the onset and progression of AF according to research in animal models and large population‐based studies [34, 35]. Some observational studies have discovered variations in gut microorganisms in patients with AF [13, 14, 36, 37, 38, 39, 40]. These alterations encompass significant species richness and diversity, an evident elevation in opportunistic pathogenic bacteria, and an obvious decline in symbiotic bacteria. These alterations in the gut microbiota are related to AF development and recurrence. These associations were validated in the present study. Our results suggest the protective role of 12 gut microbes and the harmful effect of three gut microbes on AF occurrence. Among these gut microbes, *Bifidobacterium* and *Odoribacter* at lower levels were associated with an increased risk of AF, which is supported by previous studies [35, 41]. The authors explored the association of the gut microbiota with prevalent AF based on a cohort of 6763 participants and replicated the results in a separate case–control population of 138 individuals. They found an underlying negative relationship between prevalent AF and *Bifidobacterium*. The abundance of *Bifidobacterium* is lower in patients with prevalent AF. *Bifidobacterium* is the most abundant intestinal bacteria and constituent of most probiotics, and it has multiple functions and benefits in human beings. *Bifidobacterium* decreases cholesterol levels in the blood by synthesising several vitamins, urgently absorbing vitamin D, and converting cholesterols into steroids. The reduction in serum lipids in animal models demonstrated this lipid‐lowering effect through supplementation of *Bifidobacterium* in mice fed a high‐fat diet. Probiotics containing *Bifidobacterium* have been proven to improve the atherogenic lipid profile [42]. *Bifidobacterium* also reduces blood pressure by sustaining the original balance in the intestinal flora, inhibiting renin release [43]. Moreover, *Bifidobacterium* is thought to be positively associated with ejection fraction and negatively correlated with the cardiac stress marker N‐terminal pro‐B‐type natriuretic peptide [44]. These beneficial functions of *Bifidobacterium* in the cardiovascular system provide some insights into how *Bifidobacterium* prevents AF. Aside from *Bifidobacterium*, this MR study further underscored the genetic effects of other gut microbiota on AF risk. No reverse causal association was identified in the reverse MR analysis.

Several studies have recently indicated that intestinal barrier damage due to gut microbiota dysbiosis may induce an inflammatory response, leading to the remodelling of atrial electrical activity and structure, influencing AF onset and development [45, 46, 47]. This view raises the question of whether the inflammatory response exerts a mediating effect on the causality between the gut microbiota and AF. Based on the confirmed causality between the gut microbiota and AF, we explored the causal relationship of the gut microbiota with inflammatory cytokines and the correlation between inflammatory cytokines and AF, respectively, which was an indispensable process before performing the MR mediation analysis. The results identified intestinal microbiota and inflammatory factors with significant causal relationships. Meanwhile, some inflammatory cytokines that were causally associated with AF were also identified in the present study. Inflammatory cytokines that prevented AF included the CD40L receptor, Fms‐related tyrosine kinase 3 ligand, interleukin‐6, interleukin‐7, leukaemia inhibitory factor receptor, sulfotransferase 1A1 and tumour necrosis factor ligand superfamily member 12. Inflammatory cytokines that increased AF risk included fibroblast growth factor 5, interleukin‐2 receptor subunit β and tumour necrosis factor. Furthermore, this study revealed the mediating effect of interleukin‐18 between the genus *Lachnospiraceae* (FCS020 group) (id: 11314) and AF. This MR mediation analysis demonstrated the indirect impact of gut inflammation on the heart axis in the context of AF.

A previous study reported that patients with gut microbiota dysbiosis are susceptible to AF because lipopolysaccharides, as intestinal flora metabolites, activate the NLRP3 inflammasome [35]. The role of the NLRP3 inflammasome in the pathogenesis of AF was evaluated by Zhang et al. The authors transplanted faecal microbiota from aged AF rats to young rats and detected increased expression of transforming growth factor‐β1 and α‐smooth muscle actin in the atria [17]. This effect was derived from triggering of the atrial NLRP3 inflammasome through the Toll‐like receptor 4 (TLR4)/MyD88/nuclear factor (NF)‐kB pathway. NLRP3‐targeted inhibitors were considered to revert fibrosis by decreasing the release of interleukin‐1 and interleukin‐18 [46, 47, 48, 49]. This evidence indirectly supports the interleukin‐18‐mediating effect on the causal relationship between the gut microbiota and AF in this study. We suggest one potential mechanism through which gut inflammation leads to AF, whereby activation of the NLRP3 inflammasome due to gut microbiota dysfunction stimulates interleukin‐18 secretion, in turn elevating AF risk. However, this is only speculation, and experimental trials will be needed to evaluate this hypothesis. Aside from NLRP3, several signalling pathways have been discovered between inflammatory cytokines generated by the intestinal flora and AF susceptibility, including NF‐kB and TLR4, among others. In the past, a decrease in the deficiency of enterocyte microvilli and the size of intestinal mucosal barrier proteins was observed in older and obese microbiota [17, 44]. Researchers have attributed AF in older patients and patients with obesity to microbiota dysbiosis and systemic inflammation. When encountering lipopolysaccharides stimulated by gut microbiota dysbiosis, the paracrine activity of inflammatory cytokines or organs probably creates endotoxemia, which may lead to atrial remodelling and AF development [45]. The intestinal barrier is destructed by gut microbiota dysfunction, urgently circulating toxic bacterial metabolites and burdening the inflammatory response to the circulatory system. The abnormal environment of inflammation can disturb ectopic firing, acting as a substrate for re‐entry, leading to AF. The gut microbiota utilise inflammatory factors and directly promote AF development. Some researchers have transplanted faecal microbiota in mice to explore the effects of disordered gut microbiota on AF development. Interleukin‐1β, interleukin‐6 and interleukin‐18 expression were increased in mice with faecal microbiota transplantation‐AF, while the expression of silent information regulator 1 (SIRT1) as an anti‐inflammatory mediator was decreased. This deficiency in SIRT1 activated inflammation and inflammatory mediators. Inflammation is at the center of AF pathogenesis and is strongly correlated with fibrosis. Researchers reported that disordered gut microbiota enhanced the susceptibility to AF through the microbiota–intestinal barrier–atria axis. Therefore, upregulation of inflammatory signalling pathways may participate in the pathogenesis of AF. Aside from the inflammatory pathways reported in the literature, many other inflammatory pathways may be induced by disordered gut microbiota, leading to AF development, which is a topic worthy of further exploration in the future [50].

### 4.1 Limitations

This study has some limitations. The inflammatory cytokine‐mediated causal association between the gut microbiota and AF was derived from genetic proxies of the gut microbiota. Therefore, the causal relationship identified in this study requires further validation in future studies.

## 5 Conclusion

This study is the first MR mediation analysis to present causality among the gut microbiota, inflammatory cytokines, and AF. In particular, we showed that inflammatory cytokines mediate the influence of the gut microbiota on AF risk. The genetic results of this study reveal the potential mechanisms underlying the association between the gut microbiota and AF. Furthermore, we suggest that future management interventions for AF should focus on sustaining the gut microbiota and co‐regulating inflammatory cytokines.

## Author Contributions

**Jun Chen:** conceptualization (equal), data curation (equal), investigation (equal), validation (equal), visualization (equal), writing – original draft (equal). **Yucheng Wang:** data curation (equal), formal analysis (equal). **Kangnan Wang:** revising and editing (equal), formal analysis (equal). **Ziwei Mei:** formal analysis (equal), investigation (equal), methodology (equal). **Lihong Wang:** conceptualization (equal), writing – review and editing (equal).

## Ethics Statement

Our research was conducted entirely on publicly available, anonymized data. Therefore, individual patient consent was not required. All methods followed relevant guidelines to protect the patient's privacy.

## Consent

The authors have nothing to report.

## Conflicts of Interest

The authors declare no conflicts of interest.

## Supporting information


Table S1.

## Data Availability

The datasets generated and analysed during the current study are available from the GWAS database.
